# Increased hippocampal excitability and impaired spatial memory function in mice lacking VGLUT2 selectively in neurons defined by tyrosine hydroxylase promoter activity

**DOI:** 10.1007/s00429-014-0778-9

**Published:** 2014-05-07

**Authors:** Karin Nordenankar, Casey J. A. Smith-Anttila, Nadine Schweizer, Thomas Viereckel, Carolina Birgner, Jana Mejia-Toiber, Marisela Morales, Richardson N. Leao, Åsa Wallén-Mackenzie

**Affiliations:** 1 Unit of Functional Neurobiology and Unit of Developmental Genetics, Biomedical Center, Department of Neuroscience, Uppsala University, Box 593, S-751 24 Uppsala, Sweden; 2 National Institute on Drug Abuse, Intramural Research Program, Neuronal Networks Section, 251 Bayview Boulevard, Baltimore, MD 21224 USA; 3 Brain Institute, Federal University of Rio Grande do Norte, Natal/RN, 2155 - 59056-450 Brazil; 4 Unit of Developmental Genetics, Biomedical Center, Department of Neuroscience, Uppsala University, Box 593, S-751 24 Uppsala, Sweden

**Keywords:** Reward, Oscillations, Development, Midbrain, Mouse genetics

## Abstract

Three populations of neurons expressing the *vesicular glutamate transporter 2* (*Vglut2*) were recently described in the A10 area of the mouse midbrain, of which two populations were shown to express the gene encoding, the rate-limiting enzyme for catecholamine synthesis, *tyrosine hydroxylase* (*TH*).One of these populations (“*TH*–*Vglut2 Class1*”) also expressed the *dopamine transporter* (*DAT*) gene while one did not (“*TH*–*Vglut2 Class2*”), and the remaining population did not express *TH* at all (“*Vglut2*-*only*”). *TH* is known to be expressed by a promoter which shows two phases of activation, a transient one early during embryonal development, and a later one which gives rise to stable endogenous expression of the TH gene. The transient phase is, however, not specific to catecholaminergic neurons, a feature taken to advantage here as it enabled *Vglut2* gene targeting within all three A10 populations expressing this gene, thus creating a new conditional knockout. These knockout mice showed impairment in spatial memory function. Electrophysiological analyses revealed a profound alteration of oscillatory activity in the CA3 region of the hippocampus. In addition to identifying a novel role for *Vglut2* in hippocampus function, this study points to the need for improved genetic tools for targeting of the diversity of subpopulations of the A10 area.

**Electronic supplementary material:**

The online version of this article (doi:10.1007/s00429-014-0778-9) contains supplementary material, which is available to authorized users.

## Introduction

The A10 area of the ventral midbrain is the home of the classical dopamine (DA) neurons that are important for motivation, reward, learning and memory via substantial projections into limbic and cognitive regions. A10 consists of the ventral tegmental area (VTA) and three midline nuclei, known as the interfascicular (IF), rostral linear (RLi) and caudal linear (CLi) nuclei, respectively (Fields et al. 2007; Ikemoto 2007). Recent studies have shown that the medial VTA together with IF and RLi, which collectively often are referred to simply as the VTA, are more heterogeneous in terms of cell populations than previously thought (Li et al. 2013). For example, three distinct neuronal populations expressing the vesicular glutamate transporter 2 (*Vglut2*, aka *Slc17A6*) gene, which confers a glutamatergic phenotype (Fremeau et al. 2004), have been identified within the VTA of the adult rat and mouse. Of these three VTA *Vglut2*-expressing (VTA-VGLUT2) populations, one appears to consist of purely glutamatergic neurons (“*Glu*-*only*” or “*Vglut2*-*only*”) that show similar electrophysiological properties and projections as the neighbouring DA neurons (Hnasko et al. 2012; Kawano et al. 2006; Yamaguchi et al. 2007). The other two populations both express *tyrosine hydroxylase* (*TH*), the rate-limiting enzyme in DA synthesis, along with *Vglut2*, but differ with regards to their expression of the *dopamine transporter* (*DAT*) (Li et al. 2013). Thus, the neurons in one VTA-VGLUT2 population coexpress *TH* and *DAT* and have been named “*TH*–*Vglut2 Class 1”* neurons, while the other population contains neurons that do not express *DAT* and are referred to as “*TH*–*Vglut2 Class* 2” neurons (Li et al. 2013). Both of these *TH*–*Vglut2* populations presumably represent DA neurons that corelease glutamate [reviewed in (El Mestikawy et al. 2011; Hnasko and Edwards 2012)]. In addition to the unique property of expression in DA neurons, *Vglut2* is distinct from its sister molecules *Vglut1* and *Vglut3* in that *Vglut2* is the only isoform which can be broadly detected already at midgestation, including within the developing VTA (Birgner et al. 2010). The functional role of *Vglut2* expression in DA neurons has been investigated by different laboratories using the Cre-LoxP-based conditional knockout technique (Wallén-Mackenzie et al. 2010) targeting DA neurons via DAT promoter-driven expression of Cre recombinase. Briefly summarized, these studies demonstrated that DAT-Cre-mediated deletion of *Vglut2* expression left motivation and memory parameters intact, while causing altered responses to sweet food and psychostimulants, thereby revealing a role for the glutamate-DA cophenotype in certain aspects of reward processing (Alsiö et al. 2011; Birgner et al. 2010; Fortin et al. 2012; Hnasko et al. 2010). Spatially, DAT-Cre is to date the most appropriate Cre-driver for targeting gene expression in DA neurons, but temporally, *Vglut2* expression, detected in the VTA at embryonic day (E) 11, is more tuned with *TH* expression (E11; Zetterström et al. (1997)) which precedes that of *DAT* (E14; Fauchey et al. (2000)) by several days. To explore the possibility of targeting *Vglut2* in DA neurons already from midgestation, we therefore turned to a previously validated *TH*-*ires*-*Cre* knock-in mouse (Lindeberg et al. 2004) known to recapitulate endogenous TH expression, and we could indeed verify this expected Cre activity. Somewhat surprisingly, ectopic Cre activity was also detected in the “*Vglut2*-*only*” neurons of the VTA. Thus, based on the promiscuity of the TH promoter, we hypothesized that we had a genetic tool at hand which we could use for targeting neurons within all three VTA-VGLUT2 populations, i.e. “*Vglut2*-*only”*, “*TH*–*Vglut2 Class 1”* and “*TH*–*Vglut2 Class 2”* neurons. Upon verification, we used behavioural, electrochemical and electrophysiological techniques to analyse this new conditional *Vglut2* knockout mouse.

## Materials and methods

### Animal housing

All mice used in the study were housed in the animal facility at the BMC, Uppsala University, in accordance with the Swedish regulation guidelines (Animal Welfare Act SFS 1998:56) and European Union legislation (Convention ETS123 and Directive 2010/63/EU). Ethical approval was obtained from the Uppsala Animal Ethical Committee. The animals were housed by sex in standard Makrolon cages (59 × 38 × 20 cm) with aspen wood bedding (Scanbur AB Sollentuna, Sweden) and a wooden house. The temperature was kept at 21–22 °C with a humidity of 45–55 %. A 12 h light/12 h dark cycle was used, with lights on at 07.00 h. The animals had ad libitum access to food (R36, Labfor, Lactamin, Vadstena, Sweden) and water.

### Generation of transgenic mice

The *Vglut2* ^*f/f;TH*-*Cre*^ mouse line was produced using the breeding procedure established for conditional knockout mice to ensure identical genetic background (Crusio 2004) by breeding the *Th*-*IRES*-*Cre* (here abbreviated as TH-Cre) knock-in mouse line (on a C57BL/6 J genetic background) (Lindeberg et al. 2004) to the *Vglut2* ^*f/f*^ mouse line (on a C57BL/6 J-SV129 genetic background) (Wallén-Mackenzie et al. 2009) thereby generating cKO (*Vglut2* ^*f/f;TH*-*Cre*+^) and control (*Vglut2* ^*f/f;TH*-*Cre*−^) mice as littermates which allows for behavioural phenotyping and comparison between genotype groups (Wolfer et al. 2002). The generation of *Vglut2* ^*f/f;TH*-*Cre*^ mouse line was described previously and it was demonstrated that a subset of *Vglut2* ^*f/f;TH*-*Cre*^cKO mice have an itch phenotype at a late adult stage (Lagerstrom et al. 2010). None of the analyses in the current study included adult mice showing an itch phenotype. The *Vglut2* ^*f/f*^ mouse line was bred with the *Tau* ^*mGFP*^ reporter mouse line (Hippenmeyer et al. 2005) to allow histological and single-cell RT-PCR analyses with selectivity for the TH-Cre-expressing cells, thus producing the *Vglut2* ^*f/f;Tau*-*mGFP*^ mouse, which was subsequently crossed with the TH-Cre mice to produce *Vglut2* ^*f/f;TH*-*Cre;Tau*-*mGFP*^ (cKO-Cre-GFP) and *Vglut2* ^*f/*+*;TH*-*Cre;Tau*-*mGFP*^ littermate controls (Ctrl-Cre-GFP). The *Tau* ^*mGFP*^ Cre-reporter mouse allows visualization of Cre-expressing cell nuclei by detection of β-galactosidase (β-gal) protein and projections of the corresponding cells by green fluorescent protein (GFP). Littermate control mice were used in all experiments to ensure that any aberrant phenotypes were specifically dependent on the deletion of *Vglut2* expression in *TH*-*Cre*-expressing neurons. Further, the observer was blind to the genotype of the mice until the final analysis stage.

### In situ hybridization and immunohistochemistry

#### Tissue preparation

Animals were mated overnight for production of cKO-Cre-GFP and Ctrl-Cre-GFP offspring, and females were checked for a vaginal plug the following morning. Embryos were collected at embryonic (E) day 12 and 14. In the morning of E19, pups were born and staged as P0. For dissection of embryos, pregnant females were sacrificed by cervical dislocation and embryos were removed. For collection of adult brains, mice were sacrificed by cervical dislocation and brains removed. Amniotic sac (from embryos) and tails (from pups) were collected for genotyping according to protocols previously described (Wallén-Mackenzie et al. 2006). The tissue was fixed in zinc formalin (Richard-Allan Scientific, Kalamazoo, MI) for 18–24 h at room temperature before dehydration and paraffin infusion (Tissue Tek vacuum infiltration processor; Miles Scientific, Elkhart, IN). Sections (7 μm thick) were cut on a Microm microtome, attached to Superfrost slides (Menzel-Gläser, Braunschweig, Germany) and stored at 4 °C until usage. Slides were then deparaffinized in X-tra solve (MediteHistotechnic, Burgdorf, Germany) and rehydrated in ethanol/water before subsequent treatments.

#### In situ hybridization histochemistry

For paraffin in situ hybridization histochemistry, rehydrated paraffin sections were fixed for 10 min in 4 % formaldehyde, washed in phosphate-buffered saline (PBS), and treated with proteinase K (Sigma; 27 μg/ml diluted in 10 mM Tris–HCl/1 mM EDTA, pH 8.0) for 5 min. After refixation and washes in PBS, the slides were acetylated for 10 min in a mixture of 1.3 % triethanolamine (Sigma), 0.2 % acetic anhydride (Fluka, Neu-Ulm, Germany), and 0.06 % HCl diluted in water. Slides were then incubated for 30 min in PBS containing 1 % Triton X-100 (Sigma). After subsequent washes in PBS, slides were prehybridized for 2–5 h in hybridization solution without probe [50 % formamide (Fluka), 5× saline-sodium citrate (SSC), 5× Denhardt’s, 250 μg/ml yeast transfer RNA (Sigma) and 500 μg/ml sheared salmon sperm DNA (Ambion, Austin, TX, USA) diluted in water]. The probe for *Vglut2* (covering nucleotides 1,616–2,203) was diluted to 0.1–1 μg/ml in hybridization solution and heated to 80 °C. Sections were then hybridized with 100 μl of hybridization solution for 16 h at 70 °C. The next day, slides were dipped in prewarmed 5× SSC, transferred to 0.2× SSC, and incubated for 2 h at 70 °C. After one wash in 0.2× SSC at room temperature and one wash in B1 solution (0.1 M Tris–HCl, pH 7.5, and 0.15 M NaCl), sections were immuno-blocked with 10 % foetal calf serum in B1, and then incubated overnight at 4 °C with alkaline phosphatase-conjugated anti-digoxigenin Fab fragments (Roche, Mannheim, Germany) diluted 1:5,000 in B1 containing 1 % foetal calf serum. The following day, slides were washed in B1, equilibrated in B3 (0.1 M Tris–HCl, pH 9.5, 0.1 M NaCl, 50 mM MgCl2), and colour developed in a 10 % polyvinyl alcohol (Sigma) solution also containing 100 mM Tris–HCl, pH 9.5, 100 mM NaCl, 5 mM MgCl2, 0.17 % nitroblue tetrazolium (Roche), 0.17 % 5-bromo-4-chloro-3-indolyl phosphate (Roche), and 1 mM levamisole (Sigma). Staining was sufficient after 6–24 h, whereupon slides were washed in PBS and incubated overnight with primary mouse TH (Chemicon) antibody and processed as described below (omitting the boiling procedure).

#### Immunofluorescence histochemistry

For immunofluorescence histochemistry, rehydrated paraffin sections were boiled (this step was omitted in experiments of combined in situ hybridization and immunofluorescence) for 10 min in 0.1 M citric acid (VWR International, Leicestershire, UK), pH 6.0, left to cool for 20–30 min, washed in PBS, and incubated with primary mouse TH (Chemicon), rabbit β-gal (ICN/Cappel), chicken GFP (Abcam), chicken and guinea pig VGLUT2 [own production based on peptide sequences described in (Hioki et al. 2003)] via Innovagen, Lund, Sweden), rat Nestin (Dev Studies Hybridoma Bank), rabbit vesicular inhibitory amino acid transporter (VIAAT; gift from Prof Bruno Gasnier (McIntire et al. 1997; Sagne et al. 1997), mouse alpha-Internexin (Chemicon), mouse β-III-Tubulin (TUJ1; Chemicon), rabbit Synapsin (Chemicon), respectively, in PBS with 0.3 % Triton X-100 at room temperature overnight. The following day, slides were washed in PBS and incubated with Alexa fluorescent secondary antibodies (Invitrogen, San Diego, CA) diluted 1:200 in PBS with 0.3 % Triton X-100 and 10 % goat serum for 2 h at room temperature. Slides were then washed in PBS, incubated with 1 μg/ml 4′,6′-diamidino-2-phenylindole (DAPI) (Sigma), washed again, and mounted. Images were captured on a Zeiss LSM 510 Meta confocal microscope and analysed using Volocity software (Improvision).

#### Combination of *Vglut2* in situ hybridization and TH immunolabeling

Coronal free-floating sections (10, 12 or 16 µm in thickness) were processed as described previously (Wang and Morales 2008). Sections were incubated for 10 min in phosphate buffer (PB) containing 0.5 % Triton X-100, rinsed 2 × 5 min with PB, treated with 0.2 N HCl for 10 min, rinsed 2 × 5 min with PB and then acetylated in 0.25 % acetic anhydride in 0.1 M triethanolamine, pH 8.0 for 10 min. Sections were rinsed 2 × 5 min with PB, post-fixed with 4 % paraformaldehyde (PFA) for 10 min. Prior to hybridization and after a final rinse with PB, the free-floating sections were incubated in hybridization buffer (50 % formamide; 10 % dextran sulfate; 5× Denhardt’s solution; 0.62 M NaCl; 50 mM DTT; 10 mM EDTA; 20 mM PIPES, pH 6.8; 0.2 % SDS; 250 µg/ml salmon sperm DNA; 250 µg/ml tRNA) for 2 h at 55 °C. Sections collected on glass slides were dehydrated through a series of graded ethanol (50, 70 and 95 %, 5 min for each concentration). Sections were hybridized for 16 h at 55 °C in hybridization buffer containing [^35^S]- and [^33^P]-labelled single-stranded antisense or sense Vglut2 (nucleotides 317–2,357, Accession # NM_053427) probes at 10^7^ cpm/ml. Sections were treated with 4 µg/ml RNase A at 37 °C for 1 h, washed with 1× SSC, 50 % formamide at 55 °C for 1 h, and with 0.1× SSC at 68 °C for 1 h. After the last SSC wash, sections were rinsed with PB and incubated for 1 h in PBS supplemented with 4 % bovine serum albumin and 0.3 % Triton X-100. This was followed by overnight incubation at 4 °C with an anti-TH mouse monoclonal antibody (1:500, MAB 318, Millipore, Billerica, MA) for which specificity has been documented (Tagliaferro and Morales 2008). After rinsing 3 × 10 min in PB, sections were processed with an ABC kit (Vector Laboratories, Burlingame, CA). The material was incubated for 1 h at RT in a 1:200 dilution of the biotinylated secondary antibody, rinsed with PB, and incubated with avidin-biotinylated horseradish peroxidase for 1 h. Sections were rinsed and the peroxidase reaction was then developed with 0.05 % 3, 3-diaminobenzidine-4 HCl (DAB) and 0.03 % hydrogen peroxide. Free-floating sections were mounted on coated slides. Slides were dipped in Ilford K.5 nuclear tract emulsion (Polysciences, Inc., Warrington; 1:1 dilution in double distilled water) and exposed in the dark at 4 °C for 4 weeks prior to development. Slides processed for fluorescent and bright-field histochemistry were analysed using an Olympus (Tokyo, Japan) microscope with an Optigrid system (Thales, Fairport, NY) and by confocal microscopy using the Zeiss (Oberkochen, Germany) LSM 510 META system. Images were captured using Volocity software (Improvision, Lexington, MA) and captured images were auto-levelled using Adobe Photoshop software. For cell counting within the IF and RLi areas, an observer blind to the genotype of the mice (1 cKO and 1 control) counted *TH*-*expressing*, *Vglut2*-*expressing* and *TH/Vglut2*-expressing cells in 11 cKO and 9 control sections.

### Single-cell RT-PCR

Brains were obtained from P1 cKO-Cre-GFP and Ctrl-Cre-GFPmice following decapitation. A 1 mm thick coronal slice, which contained the mesencephalon was prepared under fluorescent microscope. Excess tissue was removed until only the substantia nigra pars compacta (SNc) and A10 areas, visualized by TH-Cre-driven GFP fluorescence, remained. The tissue was collected in ice-cold dissociation solution (90 mM Na_2_SO_4_, 30 mM K_2_SO_4_, 5.8 mM MgCl_2_, 0.25 mM CaCl_2_, 10 mM HEPES, 20 mM glucose, and 0.001 % phenol red, pH 7.4) then digested with papain for 20 min at 37 °C with agitation. The tissue was then triturated by several passages through glass pipettes of decreasing diameter to obtain a cell suspension (see inset in Fig. 8 for illustration of this procedure). The cells were then centrifuged through a differential gradient to eliminate dead cells and debris. Cells were plated on poly-l-lysine-coated coverslips and left to adhere for 30 min at 37 °C. The coverslips were then washed with Krebs–Ringer buffer (KRB) (140 mM NaCl, 5 mM KCl, 2 mM MgCl_2_, 2 mM CaCl_2_, 10 mM HEPES, 10 mM glucose, 6 mM sucrose, pH 7.35) to eliminate non-attached cells and in KRB during single cell collection. GFP-expressing cells were randomly collected to avoid a selection bias towards cells that express high levels of GFP. All cells were collected individually using autoclaved borosilicate patch pipettes under RNAse-free conditions; each cell was collected by applying light negative pressure to the pipette, no intracellular pipette solution was used. The content of each pipette was transferred into individual pre-chilled tubes containing a freshly prepared solution of 20 U of RNase inhibitor and 8.3 mM DTT, samples were frozen immediately on dry ice and stored at −80 °C until use. The samples were thawed on ice and the RNA converted to cDNA by reverse transcription for 1 h using 0.5 mM dNTPs mix, 1.25 μM random primers, 40 U of RNase inhibitor, 100 U of M-MLV RT (Invitrogen), 50 mM Tris-HCl, 75 mM KCl and 3 mM MgCl_2_, pH 8.3. The RT enzyme was denatured and the cDNAs stored at −80 °C until use. A first round of PCR was performed using 1.5 mM MgCl_2_, 10 pmol of each primer, 1.0 U of platinum *Taq*-DNA polymerase (Invitrogen), 20 mM Tris–HCl and 50 mM KCl pH 8.4. Thermal cycles consisted of an initial denaturation step of 94 °C for 2 min, followed by 35 cycles of 94 °C for 50 s, 55 °C for 45 s and 72 °C for 45 s. A second nested PCR was then performed as mentioned above using 10 % of the first PCR reaction as template. All PCR products were resolved on 2.5 % agarose gels. Primers were designed based upon sequences deposited in the GenBank database (www.ncbi.nlm.nih.gov/nucleotide). The *Vglut2* primers were designed around exons 4, 5 and 6 to detect both the wildtype and the knockout allele. *TH* and *DAT* mRNA expressions were also investigated. The oligonucleotides used were Vglut2: first round sense 5´-gccgctacatcatagccatc-3´ and antisense 5´-gctctctccaatgctctcctc-3´, nested sense 5´-acatggtcaacaacagcactatc-3´ and antisense 5´-ataagacaccagaagccagaaca-3´; TH: first round sense 5´-gttctcaacctgctcttctcctt-3´ and antisense 5´-ggtagcaatttcctcctttgtgt-3´, nested sense 5´-gtacaaaaccctcctcactgtctc-3´ and antisense 5´-cttgtattggaaggcaatctctg-3´; DAT: first round sense 5´-ttcactgtcatcctcatctctttc-3´ and antisense 5´-gaagctcgtcagggagttaatg-3´, nested sense 5´-gtattttgagcgtggtgtgct-3´ and antisense 5´-gatccacacagatgcctcac-3´.

### Behavioural studies

#### Spontaneous and amphetamine-induced locomotor activity

To assess spontaneous and amphetamine-induced activity, mice were placed in automated activity chambers, so-called Locoboxes, consisting of a plastic cage (55 × 55 × 22 cm) inside a ventilated and illuminated (10 lux) cabinet (Locobox, KungsbackaMät- ochReglerteknik AB) in which activity was recorded as the following parameters: corner time, horizontal locomotion, vertical locomotion (rearing) and peripheral activity. After 30 min of baseline recording, mice received an intraperitoneal injection of saline and their locomotive activity was recorded for a further 90 min. To assess their drug-induced behaviour, 2–3 days after the saline injection, the same procedure was followed, but the mice were instead given an intraperitoneal injection of 1.5 mg/kg amphetamine. Data analysis was performed using the GraphPad Prism software (GraphPad Software Inc., La Jolla, USA).

#### The radial arm maze

The radial arm maze is a hippocampus-dependent task used to record spatial working memory (WM), in which the ability of the mouse to remember the location of food-baited versus unbaited arms is measured (Meck et al. 1984). Spatial memory performance was examined in 24 cKO mice and littermate controls, all males using an eight-armed radial maze (see Fig. 5 for illustration of the radial maze and the parameters scored). The maze, elevated 45 cm above the floor, consisted of eight open arms (60 cm long and 12.5 cm wide, surrounded by inclining walls at a height of 13 cm at the centre and 3 cm at the end of the maze arms) radiating from a central compartment (30 cm in diameter). A podium (10 × 4 cm) with a recessed food plate (diameter 3 cm) was fixed at 1.5 cm from the end of each of the maze arms. Three days prior to the beginning of the experiment, the mice were schedule-fed for 6 h/day, which was reduced to 2 h/day at the start of the behavioural studies. Four of the eight arms were baited with the preferred food, R6-38, consisting of a high content of theobroma cacao. For the acquisition period, the animals were placed individually in the centre of the maze once each day for 5 days. The animals were allowed to perform for 10 min on the first trial day and thereafter the animals were allowed to remain in the apparatus until all reinforcements were obtained or until 10 min had elapsed, whichever occurred first. The same four arms were baited with a small piece of reinforcement food pellet each day. Nineteen days after the last acquisition session a retention test was performed. The same arms were baited and the mice again the same procedure was followed. The mice were manually scored for performance during the trial time. For scoring, an entry half way into an arm was defined as an arm entry. For each trial, a reference memory error (RME) was defined as a visit into an unbaited or incorrect arm. A working memory error (WME) was defined as a re-entry into an arm in which the reward was already obtained during the session. The total number of entries into each arm and the percentage of correct responses were also scored. Results were analysed using StatView 5.0 for Windows. A 2 × 2 × 6 (genotype × sex × trial days) two-way repeated-measures ANOVA was used to assess RME, WME, total number of arm entries and the percentage of correct responses obtained during trial period. Test day was analysed by Student’s *t* test.

#### High-pressure liquid chromatography (HPLC) with electrochemical detection

Brains were obtained from cKO and littermate control mice (7 Ctrl, 7cKO) following euthanasia by cervical dislocation. The brains were put in a 1 mm coronal mouse brain matrix (Ted Pella Inc, Redding, CA, USA) kept on ice and sliced. The olfactory bulb, a combination of the substantia nigra and the ventral tegmental area (VTA/SNc), hypothalamus, nucleus accumbens (NAcc), caudate putamen (CaPu), prefrontal cortex (PFC), amygdala and hippocampus were excised from the slices, weighed and stored at −80 °C. The tissue was subsequently homogenized by sonification in 0.1 M perchloric acid solution (50 mg (1.6 mM) glutathione; 1.5 g (149 mM) 70 % PCA; 100 ml H_2_O). The homogenates were centrifuged at 4 °C for 15 min at 12,000×g and the supernatant recovered for analysis of DA and its metabolites 3,4-dihydroxyphenylacetic acid (DOPAC) and homovanillic acid (HVA) content by HPLC. 40–50 µl of the supernatant was injected onto the HPLC column with the current set to 50 nA for all samples. A mobile phase (containing 55 mM sodium acetate, 0.01 mM EDTA, 1.16 mM 1-octanesulfonic acid sodium salt, 10 % methanol, pH 4) was used to separate the analytes. Chromatograms were captured using the Azure program (Kromatek, Essex, England) and the pmol values of the peaks were calculated from a standard curve for each DA, DOPAC and HVA. All statistical analyses of the data were performed using GraphPad Prism 5.0 software. Outliers were identified using the Grubbs test and excluded from the analysis. The concentration of each metabolite was compared between the cKO and littermate control mice using the Mann–Whitney *U* test.

### Electrophysiology

In vitro local field potential (LFP) recordings from hippocampus slices were performed as previously described (Leao et al. 2009). Following decapitation under isoflurane anaesthesia, brains of P18-P25 cKO and littermate control mice were removed from the skull and placed in ice-cold high-sucrose artificial cerebrospinal fluid (ACSF) (in mM: KCl, 2.49; NaH_2_PO_4_, 1.43; NaHCO_3_, 26; glucose, 10; sucrose, 252; CaCl_2_, 1; MgCl_2_, 4). A vibratome (VT1000, Leica Microsystems) was used to obtain horizontal hippocampal slices that were moved to a submerged recording chamber containing ACSF (in mM: NaCl, 124; KCl, 3.5; NaH_2_PO_4_, 1.25; MgCl_2_, 1.5; CaCl_2_, 1.5; NaHCO_3_, 30; glucose, 10), constantly bubbled with 95 % O_2_ and 5 % CO_2_ and kept at 35 °C for 1 h then maintained at room temperature. For LFP recordings, slices were transferred to an interface-type chamber and kept at 35 °C (Zelano et al. 2013). A recording glass pipette filled with ACSF was placed in the stratum radiatum of CA3. LFP signals were amplified 100× using custom-made amplifier (John Curtin School of Medical Research, Australian National University), low-pass filtered at 3 kHz and digitized at 10 kHz by a National Instruments DAQ card. Data were analysed using Matlab (Mathoworks).

## Results

### *Vglut2* expression in TH and non-TH-expressing cells in the embryonic VTA area

**Fig. 1:**
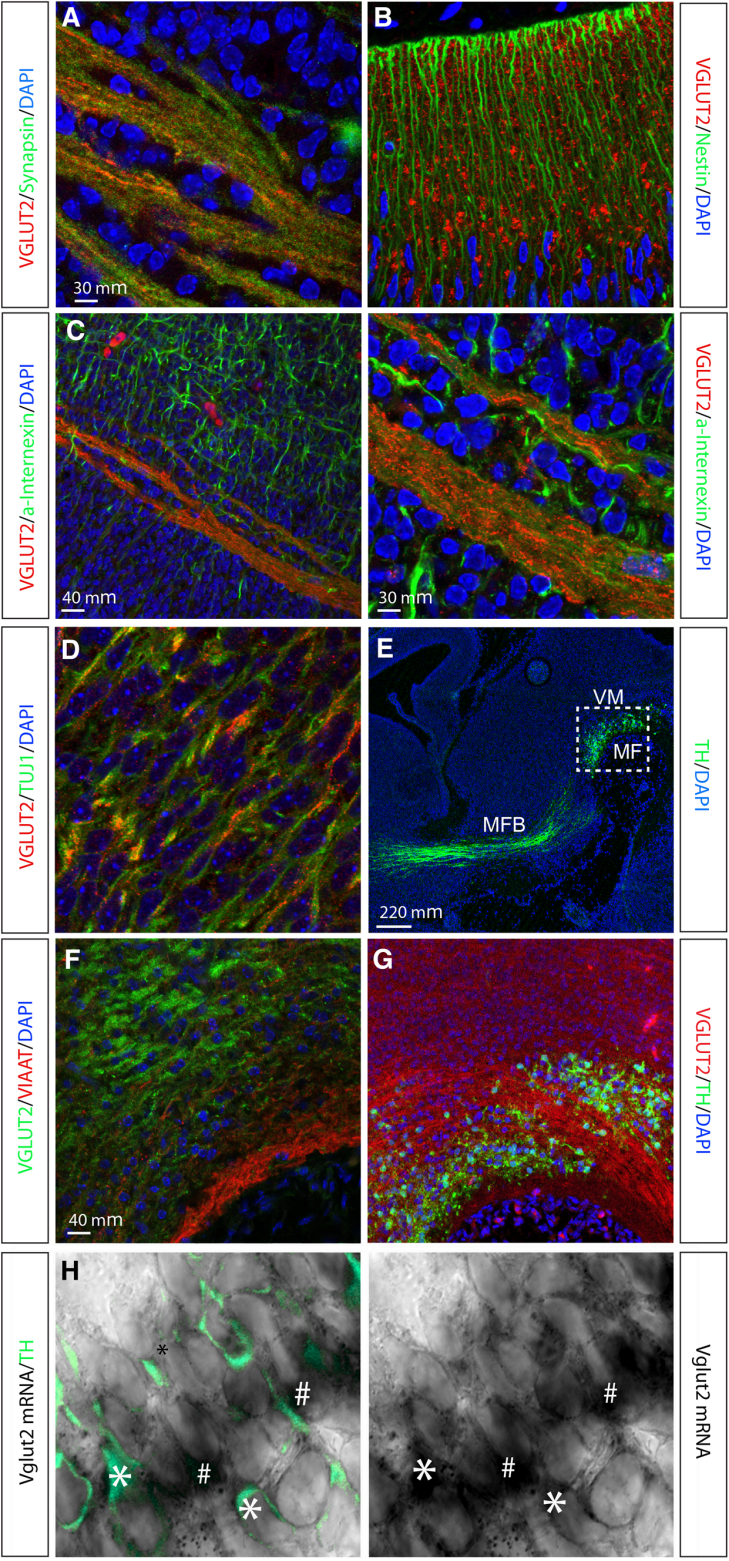
Immunohistochemical and in situ hybridization analysis on sagittal sections of the embryonal mouse brain. Immunofluorescent analysis of VGLUT2 (*red*) with cell nuclei labelled with DAPI (*blue*) at embryonic (E) day 12. Synaptic colocalization of VGLUT2 (*red*) and Synapsin (*green*) (**a**); Nestin-expressing neuronal progenitors (*green*) interspersed with VGLUT2 (*red*) (**b**); localization of VGLUT2 (*red*) within and parallel to fibrous structures immunoreactive for neuronal marker alpha-Internexin (a-Internexin; *green*) (**c** left and right); localization of vesicular VGLUT2 (*red dots*) within neuronal fibre bundles labelled by β-III-Tubulin (Tuj1; *green*) (**d**); overview (E15) of ventral midbrain (VM) to visualize the area above mesencephalic flexure (MF) and TH-positive (*green*) neurons with projections in median forebrain bundle (MFB) to striatum (**e**), *dotted square* marks the area of sections shown in **f**, **g**; VGLUT2-positive fibres (note, *green*) in the VM above the MF, no colocalization with the marker for inhibitory neurons, VIAAT (*red*) (**f**); VGLUT2-positive fibres (*red*) in VM area of TH-positive neurons (*green*) (**g**); (**h**) in situ hybridization combined with immunohistochemistry of Vglut2 mRNA (*black*) and TH protein (*green*) in the VM.Cells positive for both Vglut2 (*black*) and TH (*green*) marked with *single asterisk* cells positive for Vglut2 but not TH marked with *hash*

We have previously shown that *Vglut2* is prominently expressed in the subventricular zone in several cortical, subcortical and brainstem structures, including in *TH*-expressing neurons in the ventral midbrain, of the mouse already at midgestation (Birgner et al. 2010). To analyse if *Vglut2* gene expression at these early developmental stages results in protein products, we analysed localization of the VGLUT2 protein in combination with a range of various molecular markers by immunohistochemistry (IHC). Ample VGLUT2 protein was found in the di-, mes- and metencephalon of the developing mouse brain already at E12, detected in the distinct, dotted appearance of presynaptic structures where it colocalised with the presynaptic marker Synapsin (Fig. 1a, representative example). VGLUT2 protein did not colocalize with Nestin, a marker for dividing neural progenitor cells (Lendahl et al. 1990) (Fig. 1b), but was detected in elongated structures resembling fibre bundles, where it showed some, but not complete colocalization with alpha-Internexin (Fig. 1c), a class IV intermediate filament protein commonly used as a marker for subclasses of neurons and the general neuronal fibre marker beta-III-Tubulin (TUJ1) (Fig. 1d). These fibre structures are usually not observed when analysing VGLUT2 protein immunoreactivity in the adult brain, where it is exclusively presynaptic and vesicular, suggesting that VGLUT2 is present in the embryo already during the establishment of the developing axonal structures. When analysing the ventral midbrain (Fig. 1e for localization of area), VGLUT2 was found not to colocalize with VIAAT, a molecular marker for inhibitory neurons (Fig. 1f). *TH*-expressing neurons were found intermingled with VGLUT2-positive fibres, suggesting ample glutamatergic afferent input into this area (Fig. 1g). With the reports of a “Glu-only” neuronal population in the adult VTA (Hnasko et al. 2012; Kawano et al. 2006; Li et al. 2013; Yamaguchi et al. 2007), we wished to assess *Vglut2* expression in the ventral mesencephalic flexure where VTA and SNc neurons form (Wang et al. 1995; Ye et al. 1998). Vglut2 in situ hybridization analysis at E12 combined with IHC for TH showed a mixture of cells expressing both *Vglut2* and *TH* (“*TH*–*Vglut2*”), cells expressing *Vglut2* but not *TH* (“*Vglut2*-*only*”), and cells expressing *TH* but not *Vglut2* (“*TH*-*only*”) at all three embryonal stages examined (Fig. 1h for representative example, and Fig. 8 for illustration of these populations). Together, these analyses show that the *Vglut2* gene is expressed from midgestation and onwards in the developing mouse brain, including the developing VTA area where it is found in both *TH*-expressing and non-*TH*-expressing cells.

### TH-Cre activity detected in “TH-only”, “TH–Vglut2” and “Vglut2-only” VTA neurons

**Fig. 2:**
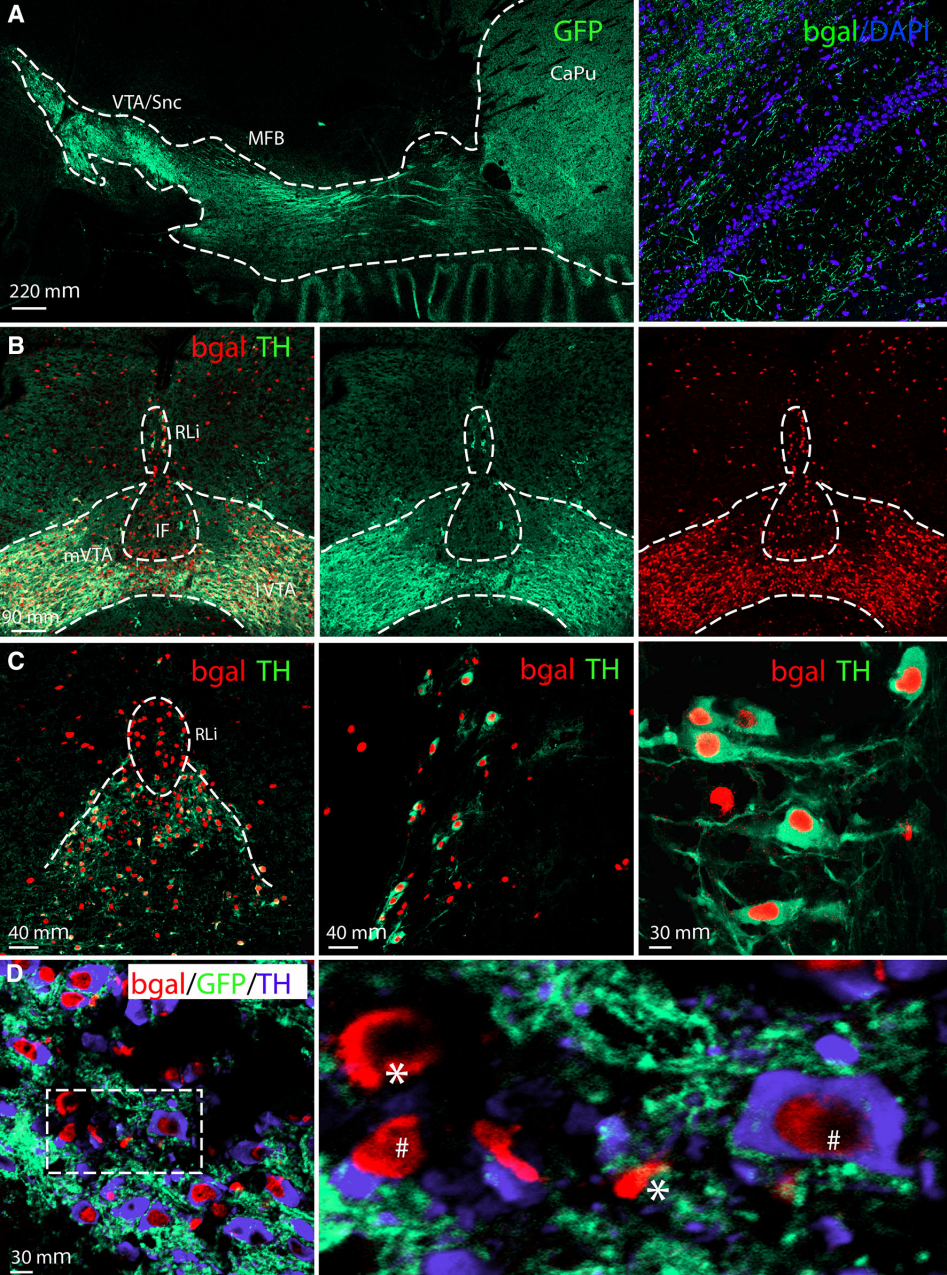
TH-Cre transgene analysis in the adult mouse brain via the tau-mGFP double Cre-reporter. Cell nuclei marked with DAPI (*blue*) (** a**). Projections from the ventral tegmental area and substantia nigra pars compacta (VTA/SNc) to the entire striatal area (*left*) via the median forebrain bundle (MFB) visualized by immunofluorescence for the green fluorescent protein (GFP), close-up of GFP fibres in the hippocampus (*right*) (**a**). Nuclear β-galactosidase (β-gal; *red*) shows the position of cells in which TH-Cre activity has enabled β-gal expression; endogenous TH protein expression is indicated by TH immunoreactivity (*green*); co-localization (β-gal (*red*) nuclei in centre of TH (*green*) cytoplasm) is mainly seen in medial (m) and lateral (l) VTA, less so in the medially located rostral linear nucleus (RLi) and Interfascicular nucleus (IF) where many cells show β-gal (*red*) only (**b**, **c**). Triple β-gal (*red*)/TH (*blue*)/GFP (*green*) immunofluorescence in RLi shows some β-gal/GFP-positive TH-Cre-reporting cells that immunopositive for TH (*hash*) and some that do not have TH (*single asterisk*) (**d** left and close-up to the right)

**Fig. 3:**
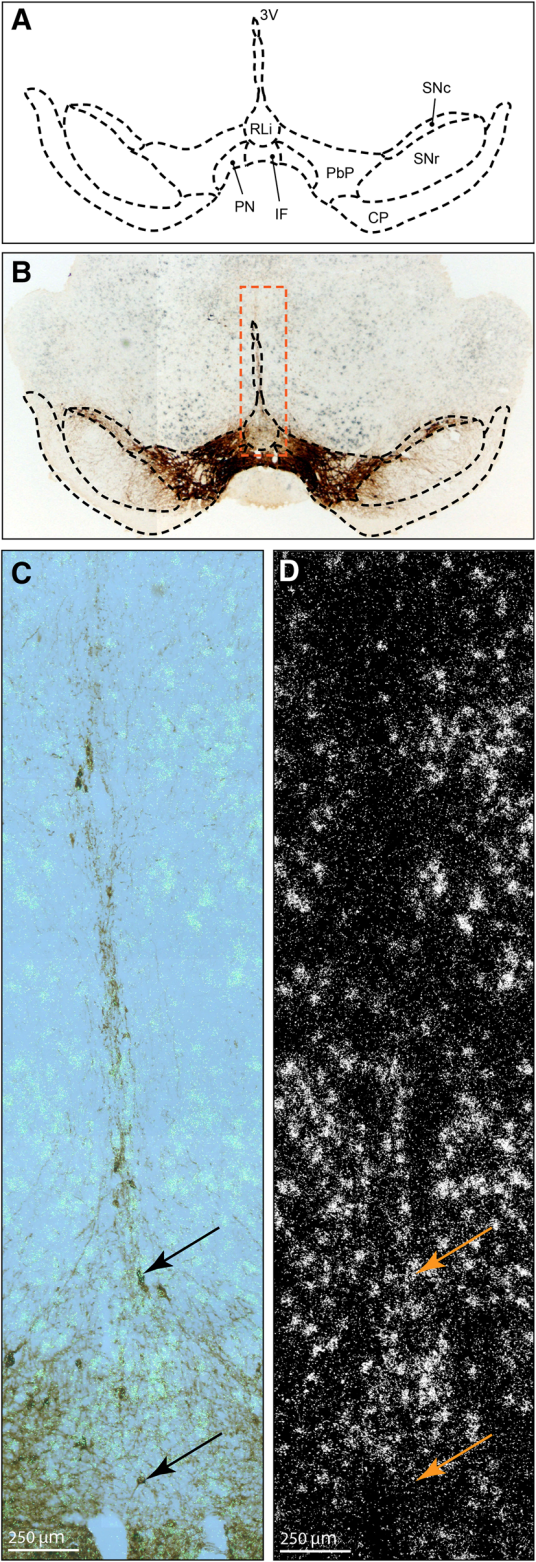
Localization of TH protein and Vglut2 mRNA by combined immunohistochemistry and in situ hybridization in coronal section of the adult mouse midbrain. Representative section at Bregma −3.16. Illustration of anatomically distinct A10 areas, including the rostral linear nucleus of the raphé nuclei (RLi); paranigral nucleus (PN); interfascicular nucleus (IF); parabrachial pigmented nucleus (PbP); also marked is the substantia nigra pars compacta (SNc); substantia nigra pars reticulata (SNr). The cerebral peduncle (CP) and third ventricle (3 V) were added as landmarks** a**; Overview of ventral midbrain showing Vglut2 mRNA broadly dispersed (*grey*) and TH-immunoreactivity (*brown*) in A10 and SNc areas, with the above outlined structures as overlay (*black dotted outline*) and indicating the area of the close-ups below (*orange dotted line*) (**b**). Close-up images of the RLi and IF as indicated in B (*orange outline*) showing TH-immunoreactivity (*brown*) and Vglut2 mRNA (*light green*) (**c**) and Vglut2 mRNA detected as silver grains (**d**). *Arrows* denote the same neurons in both panels; the *upper arrow* indicating a neuron positive for both TH and Vglut2, the *lower arrow* indicating a neuron positive for TH only

The tau-mGFP floxed Cre-reporter (Hippenmeyer et al. 2005) was used to enable validation of Cre activity in the previously described TH-Cre knock-in mouse (Lindeberg et al. 2004) by IHC for β-galactosidase (β-gal) and green fluorescent protein (GFP) in the same sections to detect TH-Cre-expressing cell nuclei (β-gal) and projections(GFP) throughout the adult brain. By analysis of Ctrl-Cre-GFP mice (see Materials and Methods for description), we could confirm the previous characterization of the TH-Cre mice (Lindeberg et al. 2004) which, using the loxP-STOP-loxP-LacZ (Tsien et al. 1996), showed TH-Cre-activity in all catecholaminergic cell groups, including the noradrenergic neurons of the A1, A2 and locus coeruleus groups, the adrenergic neurons of the C1, C2 and C3 groups in the medulla, in addition to the DA cell groups in the ventral midbrain (A8, A9, A10) and hypothalamus (A11-13) (data not shown). We could also confirm previous observations of TH-Cre activity in the dorsal root ganglia (Lagerström et al. 2010; Lindeberg et al. 2004), but in contrast to the previous characterization (Lindeberg et al. 2004), we did not detect TH-Cre activity, assessed by β-gal-IHC, in the cortex or hippocampus (data not shown). However, as described below, the hippocampus did contain GFP-immunoreactive fibres, confirming innervation from TH-Cre-expressing neurons located elsewhere (shown in Fig. 2b and also in Fig. 7a). We then focused our analyses on the ventral midbrain. By analysing GFP IHC in sagittal sections, we detected median forebrain bundle projections reaching from the VTA/SNc area to the dorsal and ventral striatum (Fig. 2a). Combined β-gal (“reporting” TH-Cre transgene activity) and TH (endogenous TH protein) IHC on coronal sections visualized the two markers in the A10 area (2C-D), with high-level magnification showing β-gal in the nuclei of TH-immunoreactive neurons (Fig. 2c–e). In addition, in the IF and RLi areas, where we (Fig. 3) and others (Hnasko et al. 2012; Li et al. 2013) detected *Vglut2*-expressing cells that did not express endogenous TH, several β-gal-positive cell nuclei were detected that lacked TH-immunoreactivity (Fig. 2b–d). Although somewhat surprising, these findings are in accordance with the original characterization of the TH-Cre transgene (Lindeberg et al. 2004)which found that due to early and transient expression from the TH promoter during development, the Cre transgene will be active early on in cells not expressing stable endogenous TH later in life (see Fig. 8 for illustration of the TH promoter activity). This seemingly ectopic Cre activity will give rise to early deletion of any floxed alleles present and which will remain gene targeted throughout their life, thereby providing a molecular mechanism for how adult A10 cells can be immunopositive for β-gal but not TH. Based on this finding, we hypothesized that mice expressing both TH-Cre and the floxed *Vglut2* allele, i.e. *Vglut2* ^*f/f;TH*-*Cre*+^ mice, should have *Vglut2* gene targeted in both “*TH*–*Vglut2*” cells and in “*Vglut2*-*only*” cells.

### Vglut2 mRNA in the adult VTA area verified in TH- and non-TH-expressing cells

Previous studies have identified highest ventral midbrain *Vglut2* expression in DA neurons of the medial A10 area, including the IF and RLi (see Fig. 3a for illustration) of the adult mouse and rat, intermingled with *Vglut2*-*only* expressing cells (Hnasko et al. 2012; Li et al. 2013; Yamaguchi et al. 2007). Based on these studies, we addressed *Vglut2* expression in the ventral midbrain of the adult mouse of C57/BL6-Sv129 mixed genetic background and could confirm these previous reports (Hnasko et al. 2012; Li et al. 2013; Yamaguchi et al. 2007). *Vglut2* in situ hybridization combined with IHC for TH showed a mixture of cells expressing both *Vglut2* and *TH* (“*TH*–*Vglut2*”), cells expressing *Vglut2* but not *TH* (“*Vglut2*-*only*”), and cells expressing *TH* but not *Vglut2* (“*TH*-*only*”, presumably classical DA neurons) in the adult A10 area (Fig. 3b–d). We next counted the number of *TH*-expressing (*Mean/SEM IF: Ctrl 33.6/8.6; cKO 34.3/10.0, RLi: Ctrl 10.9/4.8; cKO 5.1;1.2*), *Vglut2*-expressing (*Mean/SEM IF: Ctrl 8.3/3.4; cKO 4.1/1.1, RLi: Ctrl 29.0/8.6; cKO 16.5/3.3*) and *TH/Vglut2*-expressing (*Mean/SEM IF: Ctrl 0.3/0.2; cKO 0.3/0.1, RLi: Ctrl 1.8/0.5; cKO 2/1.4*) cells, respectively, in the IF and RLi areas of control (9 sections) and cKO (11 sections) mice in the *Vglut2* ^*f/f;TH*-*Cre*^ mouse line, but due to loss of material failed to reach a statistically relevant evaluation.

### The TH-Cre transgene mediates deletion in “TH–Vglut2” and “Vglut2-only” neurons

**Fig. 4:**
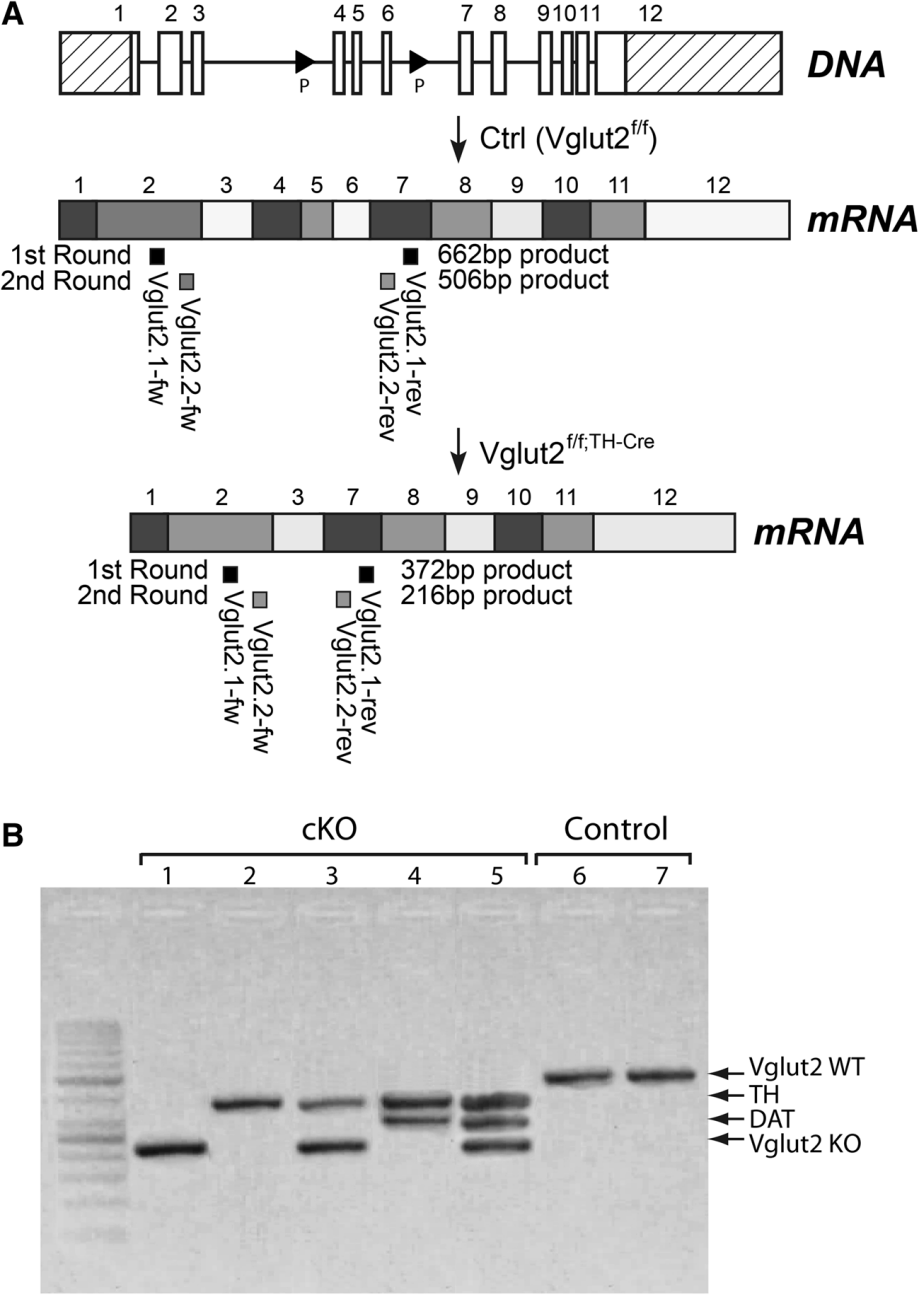
Targeting construct and single-cell RT-PCR analysis. **a** Illustration of the genomic DNA sequence showing the Vglut2 locus in a wildtype mouse (*uppermost panel*), followed by the mRNA generated from the wildtype (WT) floxed allele that has not seen TH-Cre activity (*middle*), and the mRNA of the conditional knockout allele generated after TH-Cre-activity has excised the DNA sequence between the LoxP (P) sites (*lower panel*). The *Vglut2* primers for RT-PCR analysis on single cell material were designed around exons 4 and 6 to detect both the wildtype and knockout (KO) allele, the primer annealing sites for nested RT-PCR are indicated below the transcript as follows: first round in middle panel, second round in lower panel. **b** Representative example of a gel picture showing final RT-PCR products in single cell material (derived from GFP fluorescent, dissected VTA/SNc P1 conditional knockout (cKO) and control brains, resp.);*Vglut2* (WT allele 506 bp, *uppermost band*; KO allele 372 bp, *lowermost band*), *TH* and *DAT*, all as indicated to the right of the picture

To further analyse gene expression in TH-Cre-active cells, we turned to single-cell RT-PCR experiments in freshly dissociated GFP-positive cells derived from the dissected VTA/SNc area using *Vglut2* primers designed to distinguish between the wildtype and knockout alleles (Fig. 4a). Sixty GFP-expressing cells were collected and prepared from P1 Ctrl-Cre-GFP and cKO-Cre-GFP mice, respectively (Fig. 8 for illustration). As expected, none of the green cells picked from the Ctrl-Cre-GFP mice expressed the *Vglut2* knockout allele, instead all 12 cells expressing *Vglut2* showed the wildtype allele (representative examples shown in Fig. 4b). In contrast, *Vglut2* expression in the cKO-Cre-GFP was represented almost exclusively by the knockout allele; in 28 out of 29 (97 %) *Vglut2*-expressing cells recombination had occurred leading to a deletion of *Vglut2*, thus verifying the conditional targeting of *Vglut2* (representative examples shown in Fig. 4b). In addition, primers for endogenous TH and DAT expression were used to further characterize the GFP-expressing cells. Out of 60 cells, 48 cells (80 %) were found to be expressing endogenous *TH*. Of these 48 cells, 13 cells (22 % of all cells) were coexpressing *TH* and *DAT* but did not contain *Vglut2* (presumably classical DA neurons), 5 cells (8 % of all cells) were coexpressing *TH*, *Vglut2* and *DAT* (possibly “*TH*–*Vglut2 Class* 1” neurons) and 18 cells (30 % of all cells) were found to be coexpressing *TH* and *Vglut2* (possibly “*TH*–*Vglut2 Class 2*” neurons). Further, six GFP-expressing cells (10 %) were also identified which expressed *Vglut2* but not TH or DAT (possibly “*Vglut2*-*only*” neurons). Representative examples of RT-PCR gene products are shown in Fig. 4b.

These results show that in cells expressing the TH-Cre transgene in the VTA/SNc area, as identified by green fluorescence, *Vglut2* expression is found in “*Vglut2*-*only*” cells and in both populations of cells expressing TH, i.e. those expressing *Vglut2* together with both *TH* and *DAT*, and those expressing *Vglut2* together with *TH* only (See Fig. 8 for illustration). *Vglut2* was identified as gene targeted (knocked out) within all three populations of VTA-VGLUT2 neurons. Furthermore, the results indicate that recombination has occurred in nearly 100 % of all picked GFP-positive neurons of the mesencephalon expressing *Vglut2*. This level of recombination is in accordance with our previous characterization showing gene targeting in 74 % of the *Vglut2*-expressing cells of the dorsal root ganglia (DRG) in the same mouse line (Lagerström et al. 2010). In comparison, 82 % of DAT-Cre-expressing cells which also expressed *Vglut2* showed targeting of the Vglut2 allele in the previously characterized *Vglut2* ^*f/f;DAT*-*Cre*^ mouse line (Birgner et al. 2010).

### Behavioural analysis reveals a normal amphetamine-induced activational response but decreased spatial memory

**Fig. 5:**
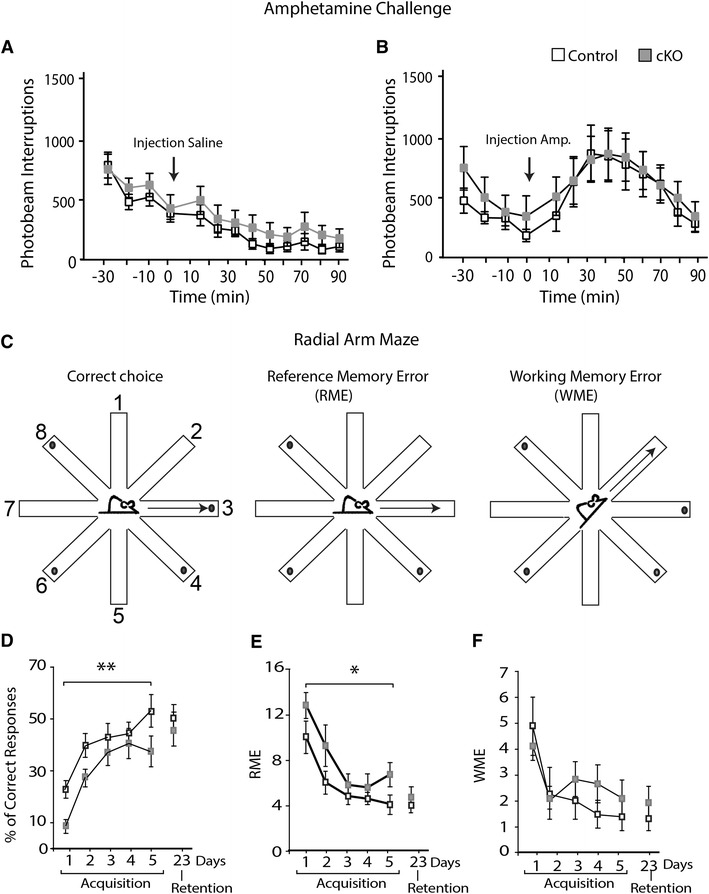
Analysis of basal and amphetamine-induced locomotion and of spatial memory. Horizontal locomotion (recorded as at least two consecutive photobeam interruptions) before and after saline- (**a**) and amphetamine- (**b**) injections; shown 30 minutes prior to injection (−30 to −10), injection indicated by time point 0, and 90 min post-injection (10–90) in 10 min intervals. Spatial memory testing in the baited radial arm maze. Schematic drawing of the maze depicting a correct choice (*left*), a reference memory error (RME) (*middle*) and a working memory error (WME) (*right*) (see materials and methods for details) (**c**). Performance of the mice during the acquisition days (1–5) and retention testing (day 23) as indicated by the number of correct choices (*left*), RMEs (*middle*) and WMEs (*right*) (**d**). *Single asterisk* *p* < 0.05; *double* *asterisk* *p* < 0.001(2-way ANOVA)

We have previously described that the *Vglut2* ^*f/f;TH*-*Cre*^cKO mice are fully viable but that TH-Cre activity in the DRG leads to an itch phenotype in some *Vglut2* ^*f/f;TH*-*Cre*^cKO mice at a late adult stage (Lagerström et al. 2010). This viable phenotype suggests absence of gross dysfunction in medullary cardiorespiratory functions, despite a presumed loss of *Vglut2* expression in C1-3 adrenal cell groups. For behavioural phenotyping of the *Vglut2* ^*f/f;TH*-*Cre*^cKO mice, we used mice around 7–8 weeks of age that did not show any itch-related phenotypes. To allow a comparison between the *Vglut2* ^*f/f;TH*-*Cre*^ and the *Vglut2* ^*f/f;DAT*-*Cre*^ mouse lines, which differ somewhat spatiotemporally in their targeting as described above, we performed the same behavioural tests of relevance to DA function as we previously performed on the *Vglut2* ^*f/f;DAT*-*Cre*^ mouse line (Birgner et al. 2010) analysing reward-related function as well as affective and cognitive function, respectively. The tests included: psychostimulant-induced activational response, the forced swim test (FST), the elevated plus maze (EPM) and the radial maze. Spontaneous and amphetamine-induced activities were recorded in activity chambers, revealing no statistically significant differences between cKO and control mice, either pre- or post-injection, in either vertical or horizontal locomotion, or the time spent in peripheral versus central areas of the chamber (Fig. 5a, b and data not shown). Further, no differences between genotypes were observed in the FST in which the cKO and control mice spent equal time swimming in a two-day trial experiment (Supplementary Fig. 1A). In the EPM, no differences between genotypes were seen in time spent, or number of entries, in any area (Supplementary Fig. 1B). Analysis of the total number of entries during the entire trial showed no differences between genotypes, suggesting that the total activity of the mice is the same for both genotypes (Supplementary Fig. 1B).

The eight-armed radial maze (illustration Fig. 5c) was utilized to determine whether there was any impairment in hippocampus-dependent spatial memory (Meck et al. 1984) performance in the cKO mice. The overall performance of the mice in the maze task was determined by the percentage of correct responses. Both the cKO and the littermate control mice increased their efficiency throughout the trials [*F*(1,100) = 12.558, *p* < 0.0001]. However, cKO mice took significantly longer to acquire the task in comparison to the littermate controls [*F*(1,20) = 8.369, *p* = 0.009] (Fig. 5d). There was no difference in the retention (18 days post-acquisition) of the learned task between the two genotypes (Fig. 5d). Reference memory error (RME) and working memory error (WME) were also investigated in the task (Fig. 5e, f). There was a significant overall difference in spatial processing throughout the trial days for both RME [*F*(5,100) = 12,234, *p* < 0.0001] and WME [*F*(5,75) = 3.645, *p* = 0.0053]. cKO mice made significantly more RMEs in comparison to the littermate control mice across the trial days [*F*(1,20) = 5.605, *p* = 0.0281] (Fig. 5e), whereas WMEs were not significantly different in the two groups [*F*(1,15) = 3.504, *p* = 0.0809] (Fig. 5f). No differences were seen between the two genotypes in either the RME or WME on day 23 (Fig. 5e, f).

Taken together, this data show that *Vglut2* ^*f/f;TH*-*Cre*^cKO mice lack alterations in depression-like and anxiety-like behaviour as measured by the FST and the EPM paradigm, respectively. These behavioural phenotypes mimic those of the *Vglut2* ^*f/f;DAT*-*Cre*^ cKO mice characterized previously (Birgner et al. 2010), as does the finding of normal levels of total activity, detected both in the activity chambers prior to injection of psychostimulant, and in the EPM. In contrast, *Vglut2* ^*f/f;TH*-*Cre*^cKO mice lack the altered response to the psychostimulant amphetamine, which is evident in the *Vglut2* ^*f/f;DAT*-*Cre*^ cKO mice, but show an impairment in the acquisition of the radial maze task and performed more spatial RMEs than control mice. Spatial memory functions are mediated by the hippocampus and were previously found normal in the *Vglut2* ^*f/f;DAT*-*Cre*^ cKO mice. Thus, the targeting of *Vglut2* expression by TH-Cre does not produce the identical phenotype as corresponding targeting by DAT-Cre, a finding likely reflected by the herein identified fact that TH-Cre and DAT-Cre differ quite substantially in their targeting of floxed *Vglut2* cells.

### HPLC analysis reveals altered DA levels in the hippocampus

**Fig. 6:**
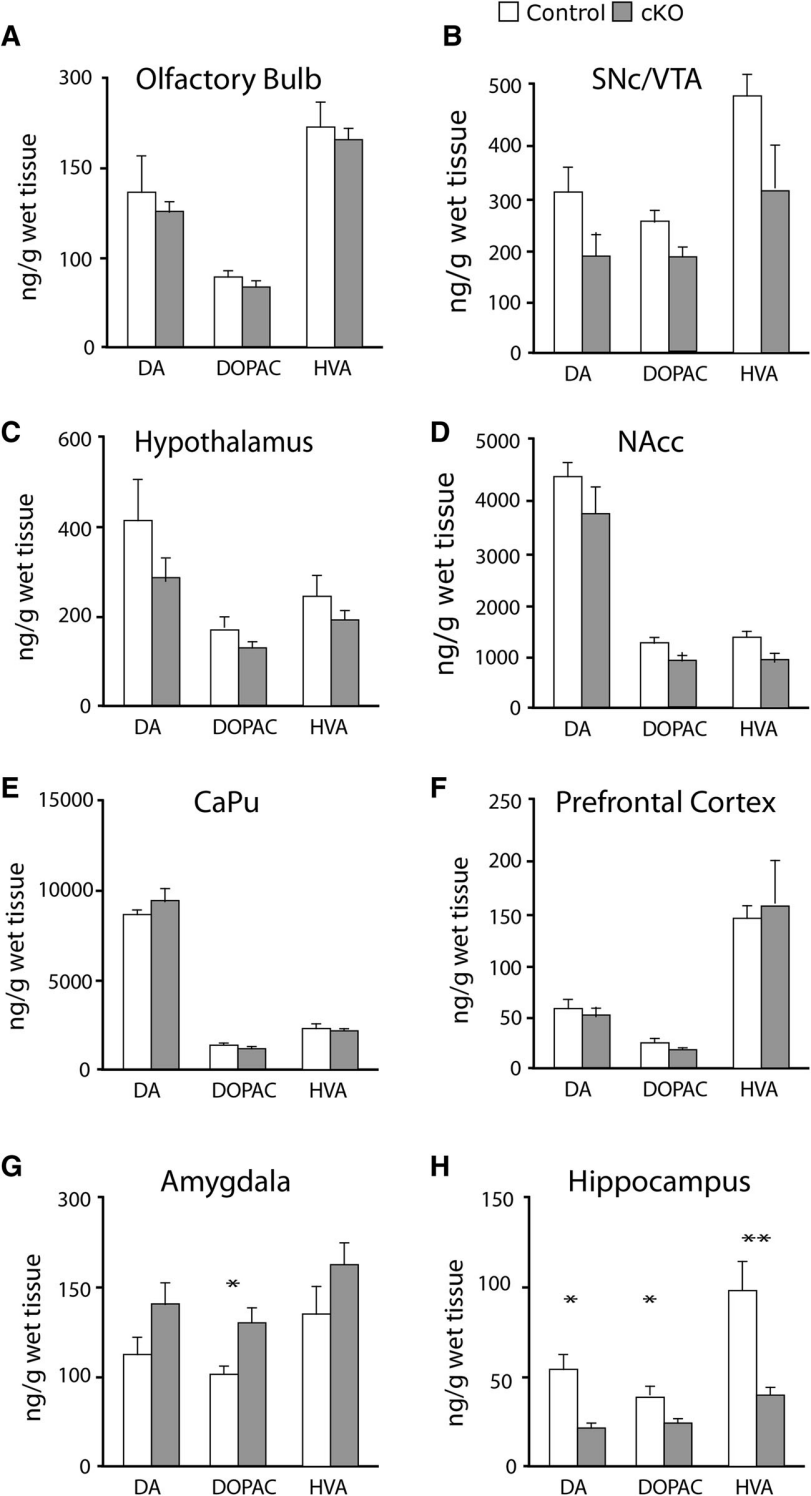
HPLC analysis of DA and its metabolites in dissected brain material. Tissue content of dopamine (DA), 3,4-Dihydroxyphenyl-acetic acid (DOPAC) and homovanillic acid (HVA) of control (*white bars*) and cKO (*grey bars*) mice, all in ng/g wet tissue. Olfactory bulb (**a**); substantia nigra pars compacta/Ventral tegmental area (SNc/VTA) (**b**); hypothalamus (**c**); nucleus accumbens (NAcc) (**d**); caudate putamen (CAPu) (**e**); prefrontal cortex (**f**); amygdala (**g**); hippocampus (**h**). *Single asterisk* *p* < 0.05; *double* *asterisk p* < 0.001 (Mann–Whitney *U* test)

The VGLUT2 protein has been suggested to play a role in DA transmission by affecting vesicular packaging of DA by a process termed “vesicular synergy”, a concept which postulates that a VGLUT may work in synergy with another vesicular transporter, such as the monoamine transporter VMAT2, to increase the efficiency of this transporter (Gras et al. 2008). We and others have previously shown that loss of VGLUT2 in DA neurons in the *Vglut2* ^*f/f;DAT*-*Cre*^ mouse line leads to decreased DA release, possibly, but not conclusively, supporting the concept of vesicular synergy between VGLUT2 and VMAT2 (Alsiö et al. 2011; Hnasko et al. 2010). To assess if the targeted loss of VGLUT2 would affect DA levels in the *Vglut2* ^*f/f;TH*-*Cre*^ mouse line, HPLC was used to assess possible differences in the concentration of DA as well as its metabolites DOPAC and HVA in cKO and control mice. This analysis was carried out in a number of different brain regions, including the VTA/SNc area, the olfactory bulb and the hypothalamus, all of which contain DA neuronal cell bodies, as well as the nucleus accumbens (NAcc), caudate putamen (CaPu), prefrontal cortex (PFC), amygdala and the hippocampus as these regions are known target areas of DA neurons. No difference was observed in DA, DOPAC or HVA concentrations in the olfactory bulb, VTA/SNc, CaPu, NAcc, hypothalamus or the PFC between cKO and littermate control mice (Fig. 6a–f). However, a significant increase was observed in DOPAC concentration in the amygdala of cKO mice in comparison to controls (*p* = 0.0426), but not in DA or HVA (Fig. 6g). In contrast, the concentration of DA (*p* = 0.0311), DOPAC (*p* = 0.0489) and HVA (*p* = 0.0022) was all significantly reduced in the hippocampus of cKO mice in comparison to littermate controls (Fig. 4h).

The observations of reduced DA tissue levels in the hippocampus support our behavioural observation of a possible alteration in the hippocampus due to the lack of *Vglut2* expression in TH-Cre active cells.

### Hippocampus slices from cKO mice show increased kainate-induced excitability

**Fig. 7:**
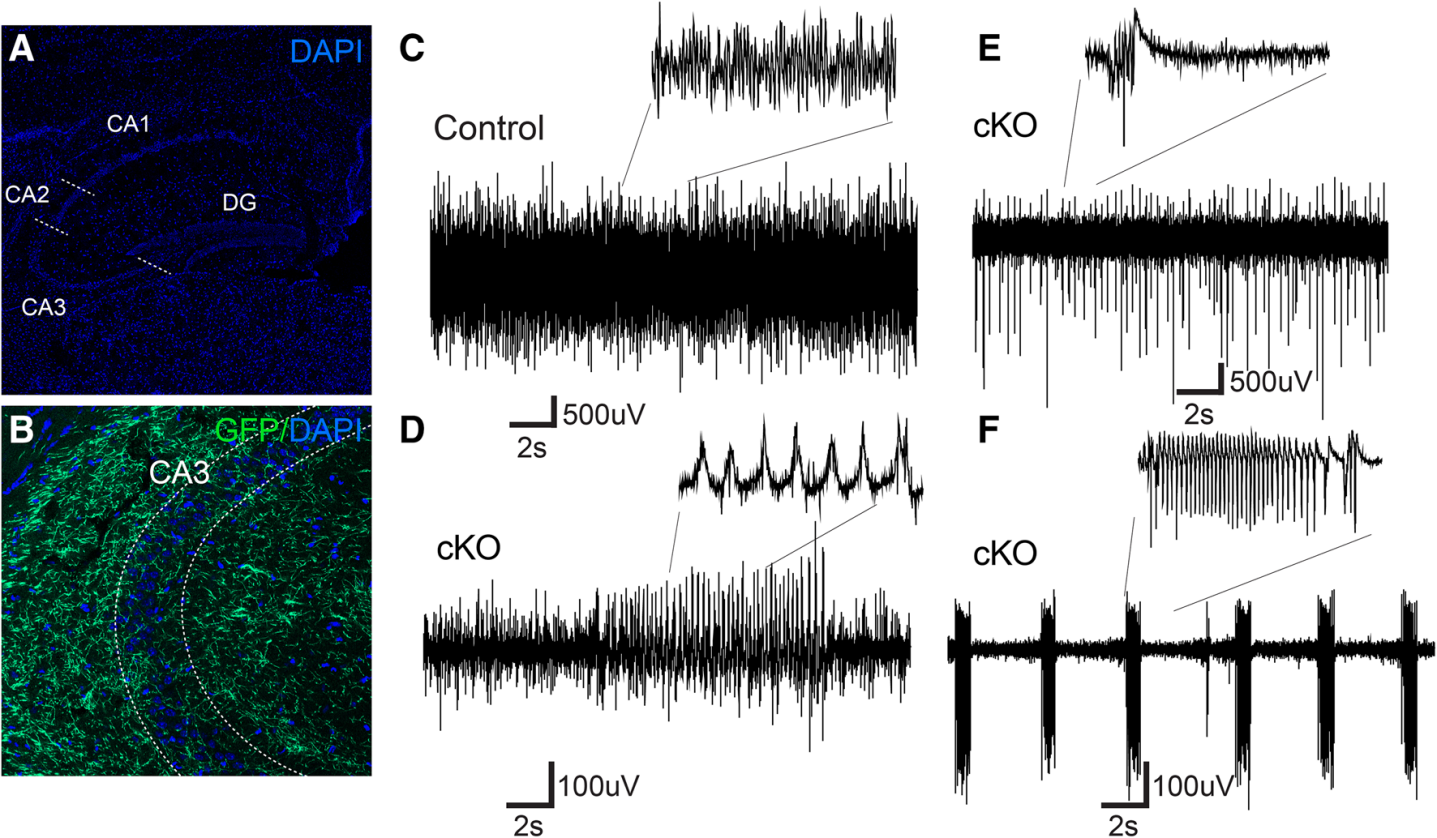
Local field potential (LFP) recordings in the CA3 of the hippocampal slice preparation. Immunofluorescent analysis of coronal hippocampal sections. Overview of hippocampus regions [CA1, CA2, CA3 and dentate gyrus (DG)] with cell bodies visualized by DAPI (*blue*) (**a**); higher magnification close-up image of the CA3 region revealing projections labelled with green fluorescent protein (GFP; *green*) from TH-Cre-reporter activity, cell bodies marked with DAPI (*blue*) (**b**). LFP recordings of the CA3 region of hippocampal slices taken from Control and cKO mice; representative example of a control slice showing stable gamma oscillations (**c**); three examples of different forms of epileptiform activity observed in CA3 slices of cKO mice (**d**–**f**)

As mentioned above, Ctrl-Cre-GFP mice show ample TH-Cre-dependent projections throughout the hippocampal formation (Fig. 7a, b), and behavioural phenotyping using the radial maze for assessment of spatial memory function suggests an impairment in the hippocampus. Signalling between the VTA and hippocampus, especially DA release in the hippocampus, has been shown to be important for the formation of long-term memory (Lisman and Grace 2005). The VTA is furthermore involved in the generation of rhythmic activity in the hippocampus (Orzeł-Gryglewska et al. 2012). We, therefore, hypothesized that the pattern of network oscillations could be altered in cKO mice. To investigate this, we recorded local field potential (LFP) in vitro in the CA3 area of the hippocampus by using horizontal slices (Leao et al. 2009). In slices derived from control mice, 100 nM kainate application produced stable gamma oscillations (7 in 8 slices from 3 animals, Fig. 7c) and epileptic-like events (Zelano et al. 2013) in one of the slices. In contrast, slices from cKO mice showed profound changes in excitability when perfused with 100 nM kainate (Fig. 7d–f). Kainate application elicited gamma oscillation in only one slice (*n* = 8 slices from 3 animals, *p* = 0.01, *Z* test) while epileptic-like events were recorded in the remaining 7 slices (*n* = 8 slices from 3 animals, *p* = 0.003, *Z* test, Fig. 7d–f).

These profound differences in CA3 responses to kainate challenge indicate an impairment of cKO mice in the ability to generate stable network oscillations.

## Discussion

The present study focused on investigating the function of *Vglut2* expression in neurons that either transiently or stably also express *TH*, the rate-limiting enzyme for catecholamine synthesis. We found that the targeted deletion of *Vglut2* in TH-expressing neurons resulted in a memory formation-deficit observed as an increased time required to acquire hippocampus-dependent spatial memory and elevated amount of errors produced in the radial arm maze, a finding which could possibly be associated with the observations of decreased DA levels and altered firing pattern in CA3 neurons of the hippocampus.

**Fig. 8:**
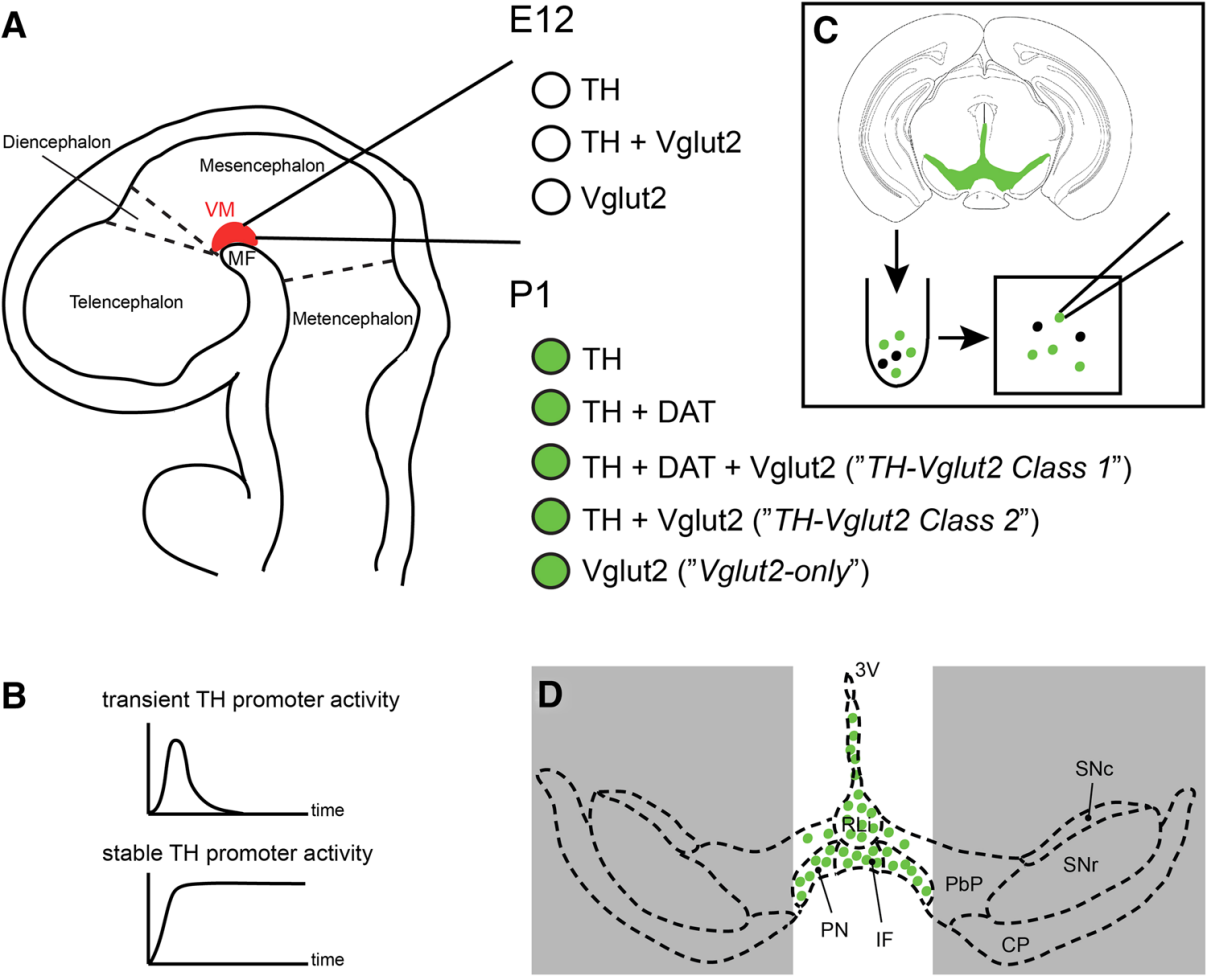
Illustration of the analysis of subpopulations in the ventral midbrain. **a** Schematic drawing of an E12 mouse embryo (seen in *sagittal view*) in which the ventral midbrain (VM) area, where the dopamine (DA) neurons develop, is highlighted in red. In our analysis (Fig. 1), we looked at the colocalization of Vglut2 mRNA with TH immunoreactivity and found cells expressing both and either of these genes, i.e. TH only, Vglut2 only and TH–Vglut2 together. At this stage, DAT is not expressed, and was therefore not analysed. **b** Representation of the two known phases of TH promoter activity, a transient phase which is not specific to cells that will develop into catecholaminergic neurons, and a stable phase which gives rise to the stable TH expression in catecholaminergic neurons. **c** Schematic drawing of the single cell selection we performed at P1 (Fig. 3) to analyse gene expression in TH-Cre cells by RT-PCR. Coronal midbrain slices of Ctrl-Cre-GFP and cKO-Cre-GFP mice, in which TH-Cre activity has led to expression of the Cre-double reporter Tau^mGFP^ thus enabling localization of these cells by GFP in fluorescent microscope, were prepared on vibratome and the entire GFP-positive ventral area [encompassing the A10 area (the rostral linear nucleus of the raphé nuclei (RLi); paranigral nucleus (PN); interfascicular nucleus (IF); medial parabrachial pigmented nucleus (PbP)] together with the lateral VTA and substantia nigra pars compacta (SNc), but not substantia nigra pars compacta (SNr) was dissected and triturated into single cell solution from which single GFP-positive cells were picked. **d** The subsequent RT-PCR analysis using Vglut2 primers (designed to recognize mRNA both from the wildtype and knockout in the same reaction), DAT primers and TH primers led us to conclude that TH-Cre is active in several populations of cells in the GFP-positive dissected area of the ventral midbrain. Based on ours (Fig. 2) and previous studies that have shown that, in the adult, *Vglut2* is most highly expressed in the A10 nuclei and not in the lateral VTA and SNc, and our observation of TH-Cre activity (detected as β-galacosidase in Fig. 3) suggest that the Vglut2 mRNA we detect is derived from the A10 nuclei. We cannot rule out that the Vglut2 we observe is derived from the lateral structures (*shadowed in grey*) that were also dissected, although this seems less likely. In summary, we found single GFP-positive cells expressing the combination of TH, Vglut2 and DAT as illustrated by the *green dots* in the figure: TH only; TH and DAT; TH and DAT and Vglut2; TH and Vglut2 but no DAT; Vglut2 only. Cells showing TH and DAT and Vglut2 have previously been termed “*TH*–*Vglut2 Class 1*” and cells showing TH and Vglut2 but no DAT have been termed “*TH*–*Vglut2 Class 2*”, a terminology we adapted and refer to in the current study. All literature referred to is listed in text

The role of glutamatergic signalling in cells that express *Vglut2* has been investigated using the Cre-LoxP-based conditional knockout technique (Wallén-Mackenzie et al. 2010). A full *Vglut2* knockout mouse, driven by the PGK-Cre transgene, resulted in dysfunctional glutamatergic transmission in the pre-Bötzinger complex causing neonatal lethality due to immediate respiratory failure, thereby demonstrating that *Vglut2* expression is necessary for post-uterine life (Wallén-Mackenzie et al. 2006). In our extended analysis of VGLUT2 at a midgestational embryonic stage (E12), we now show that this protein is found throughout the brain already at this early developmental stage, putatively supporting a broader role in establishment of the nervous system than previously recognized, but which could gain from further exploration. For gene targeting of *Vglut2* expression within DA neurons specifically, DAT-Cre transgenic mice, of which there exists a couple of variants, have been used by breeding to floxed *Vglut2* ^*f/f*^ mice, of which there also exist a few variants (Birgner et al. 2010; Fortin et al. 2012; Hnasko et al. 2010). Data have generally been interpreted based on the assumption that DAT-Cre will be active in all DA neurons and similar assumptions are common in neighbouring research fields where DAT-Cre mice are used to target other gene expression in DA neurons [e.g. the selective targeting of components of the glutamatergic machinery; reviewed in Rodriguez Parkitna and Engblom (2012)]. However, it is well known that not all *TH*-expressing neurons of the VTA area express *DAT* (Lammel et al. 2008; Sesack et al. 1998). Thus, via DAT-Cre-mediated deletion of *Vglut2* in DA neurons, which gives rise to altered responses to both sugar-rich food and psychostimulants, the so-called “*TH*–*Vglut2 Class 2*” population, which lacks *DAT* expression, has probably remained undisturbed, and the observed reward-related phenotype has been due to targeted deletion of *Vglut2* in the “*TH*–*Vglut2 Class 1*” population only. As curious as this is, the situation becomes slightly more complex when using a TH-Cre transgene, as we did here, to target *Vglut2* expression. This is brought about not only because *TH* is endogenously expressed in all catecholaminergic neurons thereby targeting also the C1, C2 and C3 groups that have been reported to also contain some *Vglut2*-expressing cells (Stornetta et al. 2002a, b), and which therefore may contribute to the behavioural phenotype, but because the TH promoter shows a complex temporal pattern of activity that gives rise to “promiscuous”, but transient, activation of the Cre recombinase also in non-catecholaminergic cells during early development (see Illustration Fig. 8). Such populations include both non-neuronal (e.g. germ cells and heart cells) and neuronal cells (e.g. the dorsal and inferior colliculus, the pontine reticulotegmental nuclei, tegmental and parabrachial nuclei, cerebellar Purkinje cells, mammillary nucleus, and also scattered cells throughout the cortex, the lateral and medial thalamus, and the striatum), i.e. both inhibitory and excitatory populations; some of which may coexpress *Vglut2*. While the normal life span of the cKO mice, as well as the normal activational level of the mice in the both the EPM and FST tests and when the mice are monitored in automated locomotor chambers, are findings suggestive of an absence of a critical role for *Vglut2* in the cardiorespiratory C1–3 neurons, the promiscuity of the TH promoter may present to be a greater challenge. Indeed, we have previously shown that activation of Cre in the DRG due to transient activation of the TH-promoter caused an itch phenotype in a subset of the cKO mice (Lagerström et al. 2010). By selecting non-itch-struck mice in the current study, we instead found a rather unexpected way to take advantage of the promiscuous TH promoter. This approach enabled, for the first time, the targeting within all three VTA-VGLUT2 populations, i.e. both populations (“*TH*–*Vglut2 Class 1*” and “*TH*–*Vglut2 Class 2*”) that express stable *TH* which can be detected as protein by IHC at any stage of life as well as the population that is characterized by absence of stable *TH* expression (“*Vglut2*-*only*”), but which, as it now turned out by the identification of the KO allele also in these cells, had experienced TH-Cre activity during early embryo stages (see Illustration in Fig. 8 for a summary of the *Vglut2*-expressing cell groups found in the ventral midbrain at E12 and P1, respectively). In situ hybridization failed to detect a significant difference between adult cKO and control mice, likely due to the low number of animals used and/or the fact that the in situ probe bound to the untargeted exons 1–3 as well as downstream targeted exons 4–6, possibly resulting in similar binding efficiency between genotypes. However, using semi-quantitative RT-PCR on single cell material, as we have also done before to verify targeting of the Vglut2 gene (Birgner et al. 2010), the KO allele was readily detected. This TH-Cre-mediated targeting of Vglut2 gave rise to a 97 % recombination efficiency in the VTA/SNc area and included all three categories of populations expressing the TH-Cre-transgene, while DAT-Cre-mediated targeting did not include the “*Vglut2*-*only”* population but efficiently targeted Vglut2 in DAT-Cre-expressing cells (82 % efficiency) in the VTA/SNc area (Birgner et al. 2010). One important caveat of the current study concerns the extent of TH-Cre activity in the three VTA subpopulations. Thus, while the TH-Cre-mediated targeting was highly efficient within cells expressing the TH-Cre transgene of each subpopulation, we cannot rule the existence of *Vglut2*-expressing cells within any of the VTA subpopulations not expressing the TH-Cre transgene; such cells would remain untargeted in this approach. Behavioural phenotyping of the new TH-Cre-mediated Vglut2 cKO mice showed the same absence of anxiety- and depression-related phenotypes as the mice carrying a DAT-Cre-mediated gene targeting of *Vglut2*, but the behavioural profile was the “opposite” when it came to amphetamine-induced psychomotor response versus hippocampus-dependent spatial memory. The lack of alteration in amphetamine-response was unexpected, given the strong phenotype of the *Vglut2* ^*f/f;DAT*-*Cre*^ cKO mice. This finding might be explained by the temporal (TH-Cre activity precedes that of DAT-Cre) or the spatial (selectivity) difference between the two Cre mouse lines (or both). For example, by carrying the potential of deleting *Vglut2* expression already from midgestation, temporally defined developmental compensations may take place in the *Vglut2* ^*f/f;TH*-*Cre*^ mice that will no longer be possible upon targeting in the *Vglut2* ^*f/f;DAT*-*Cre*^ mice, which occurs several days later. The spatially broader targeting of the TH-Cre over the DAT-Cre, the latter of which occurs only in subsets of DA cells and not at all in *Vglut2*-*only* cells, may also contribute to the difference in reward-related responses, although mechanisms remain to be established. Another important factor that has to be taken into account is the difference in genetic backgrounds of the TH-Cre (pure C57/Bl6 J background) and DAT-Cre [mixed C57BL/6 J-SV129 background (Birgner et al. 2010)] mice which, when bred to the *Vglut2* ^*f/f*^ mice (mixed C57BL/6 J-SV129 background), give rise to offspring of which the *Vglut2* ^*f/f;TH*-*Cre*^ mice will contain a higher level of C57/Bl6 J than the *Vglut2* ^*f/f;DAT*-*Cre*^ mice. Thus, both selectivity and genetic background might contribute to the observed differences in reward-related response between the *Vglut2* ^*f/f;TH*-*Cre*^ mice and *Vglut2* ^*f/f;DAT*-*Cre*^ mice.

A deeper understanding of the observed complex hippocampal phenotype will require additional approaches, but will be discussed briefly here. Taken together, three independent results point towards a hippocampus-related phenotype in the TH-Cre-mediated cKO mice; the slower acquisition and the accentuated error rate in the radial arm maze, a spatial memory test designed to assay hippocampus function (Meck et al. 1984); the significantly lower DA tissue levels observed by electrochemical detection in homogenates prepared from the hippocampus (while no other region containing either DA cell bodies or projection terminals showed similar alterations); and the increased sensitivity to the ionotropic glutamate receptor agonist kainate, leading to seizure-like activity in slice preparations obtained from cKO brains. In recent studies, it was shown in rats that severing of the signalling from the VTA to the hippocampus via the local administration of lidocaine (Mahmoodi et al. 2011) or baclofen (Martig and Mizumori 2011), resulted in a decreased performance in memory tests. Neither study showed motivational or movement disruptions, but an increase in errors and an elevated number of trials required to reach the acquisition criterion in memory tasks (Mahmoodi et al. 2011; Martig and Mizumori 2011). It could therefore be proposed that the memory deficits seen in our cKO mice may be caused by the disruption of the DA signalling between the VTA and hippocampus. Further, in a previous study, 6-hydroxydopamine lesioning of the VTA in rats resulted in the loss of approximately 50 % of DA neurons leading to a 74 % reduction in DA levels in the hippocampus as well as an 83 % loss in the frontal cortex (Wisman et al. 2008). The loss of DA was associated with a poorer reference memory performance, with repeated trials required for the rats to acquire the task (Wisman et al. 2008). These findings highlight the important role of the VTA DA system in learning and consolidation of memory. Importantly, our observation of a decreased capability of the cKO mice during the acquisition phase in the radial arm maze, and the observations we make in the seizure-prone slice preparation could also be due to a loss of glutamatergic signalling, by the targeting of “*Vglut2*-*only*” cells and the glutamatergic, instead of the dopaminergic, component of the glutamate/DA cophenotypic cells. Electrical stimulation of the VTA has previously been shown to elicit theta oscillations, important for long-range synchronization of neuronal activity, in the hippocampus (Orzeł-Gryglewska et al. 2012). Therefore, disruption of glutamatergic signalling originating from the VTA can lead to reduced activity in the hippocampal area. Known effects of activity deprivation in the hippocampus are downregulation of GABA_A_ receptors (Kilman et al. 2002) and reduction of GABA immunoreactivity, as shown in neocortical and hippocampal culture neurons (Marty et al. 1997). Maintenance of homeostasis is likely driven by a similar mechanism in the hippocampus of cKO mice. This would, as a consequence, lead to less activation of the hippocampal circuits through the VTA and an adaption of the circuitry that might already start during embryonal development. Since projections from the VTA are severed in hippocampal slice preparations, the remaining circuit is solely dependent on inhibitory and excitatory connections within the hippocampus. As a putative result of a compensatory downregulation of inhibitory components in the cKO mice upon the deprivation of VTA-derived activity, isolated hippocampal could exhibit a general overexcitation. In the presence of 100 nM kainate, a possible imbalance in the excitation/inhibition ratio could therefore lead to the generation of epileptiform discharges observed in the cKO mice, while control mice show normal gamma oscillations. Notably, given the promiscuous nature of the TH promoter as discussed above, it is conceivable that the hippocampus-related effects are independent of any alterations occurring in *Vglut2* expression in the VTA area, but are derived from alterations in *Vglut2* expression elsewhere. A broader range of genetic tools for ascertaining more selective gene targeting events, both spatially and temporally, should prove helpful in resolving the functional correlation between glutamate/dopamine transmission from the VTA and the herein described hippocampus-related phenotypes.

In summary, using a TH-Cre knock-in strategy which gives rise to Cre activity both in stable *TH*-expressing neurons and in cells that only transiently express *TH* during early development, we found a way to target *Vglut2* gene expression within all three VTA-VGLUT2 populations identified in the medial aspects of the A10 area. Both the unexpected absence of altered psychostimulant-induced behavioural activation and the identification of the strong deficiency in hippocampus function require further investigation, not least due to the promiscuity of the TH-Cre transgene which is expressed in several non-neuronal and neuronal populations in addition to the VTA. Today, most studies, even those using optogenetics to control neural activity, rely on DAT-Cre or TH-Cre for targeting of DA cells. With the accelerating gain of knowledge of various subpopulations within the A10 area, it is becoming increasingly evident that the field would benefit from a broader selection of genetic tools to enable further characterization of the physiological roles of these different neuronal groups.

## Electronic supplementary material

Below is the link to the electronic supplementary material.

Supplementary material 1 (PDF 204 kb)

## Abbreviations

β-Gal: β-Galactosidase
CaPu: Caudate putamen
cKO: Conditional knockout
CLi: Caudal linear nucleus
Ctrl: Control
DA: Dopamine
DAB: 3,3-Diaminobenzidine-4
DAPI: 4′,6′-Diamidino-2-phenylindole
DAT: Dopamine transporter
DOPAC: 3,4-Dihydroxyphenylacetic acid
DRG: Dorsal root ganglia
EPM: Elevated plus maze
FST: Forced swim test
GFP: Green fluorescent protein
HVA: Homovanillic acid
IF: Interfascicular nucleus
IHC: Immunohistochemistry
IHC: Immunohistochemistry
KRB: Krebs–Ringer buffer
LFP: Local field potential
lVTA: Later ventral tegmental area
MFB: Medial forebrain bundle
NAcc: Nucleus accumbens
PB: Phosphate buffer
PFA: Paraformaldehyde
PFC: Prefrontal cortex
RLi: Rostral linear nucleus
RME: Reference memory error
SNc: Substantia nigra pars compacta
SSC: Saline-sodium citrate
TH: Tyrosine hydroxylase
TUJ1: Beta-III-tubulin
Vglut2: Vesicular glutamate transporter 2
VIAAT: Vesicular inhibitory amino acid transporter
VTA: Ventral tegmental area
WME: Working memory error
