# Supplementary material for: Increased hippocampal excitability and impaired spatial memory function in mice lacking VGLUT2 selectively in neurons defined by tyrosine hydroxylase promoter activity
Source: Brain Struct Funct. 2014 May 7;220(4):2171–90. doi: 10.1007/s00429-014-0778-9 (PMC4481332; doi:10.1007/s00429-014-0778-9)
Supplement: Supplementary file 1 — Supplementary material 1 (PDF 204 kb) [file 429_2014_778_MOESM1_ESM.pdf]

Increased hippocampal excitability and impaired spatial memory function in mice lacking VGLUT2 selectively in tyrosine hydroxylase-expressing neurons

Brain Structure and Function

Karin Nordenankar, Casey J.A. Smith-Anttila, Nadine Schweizer, Thomas Viereckel, Carolina Birgner, Marisela Morales, Richardson Leao, Åsa Wallén-Mackenzie

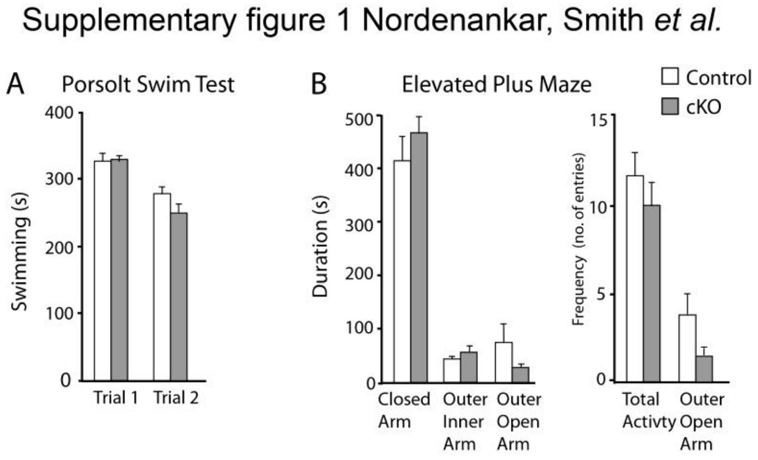

Suppl. Figure 1. ***The forced swim test (FST) and Elevated plus maze (EPM).***
(A) Depression-like behaviour of *Vglut2*$^{f/f;TH\text{-}Cre}$ cKO and littermate control mice assessed by the FST in a two-trial setting on two consecutive days. The mice were placed in a perspex cylinder filled with 30 cm deep, 25°C warm water. Each trial was filmed and lasted for 6 minutes. The time (seconds) spent swimming was scored manually by using the AniTracker software. (B) Anxiety-like behaviour was assessed in the EPM. Mice were placed in the center of the maze and allowed to explore it freely during a 10-minute trial. The latency of entrance into each arm, the time (duration) spent in each arm and the number of entries (frequency) into each area were analysed.

Corresponding author: Åsa Wallén-Mackenzie, Assoc. Prof., Department of Neuroscience, Unit of Developmental Genetics, Group of Neurocircuitry of the Basal Ganglia, Biomedical Center, P.O. Box 593, Uppsala University, S-751 24 Uppsala, Sweden.
E-mail: asa.mackenzie@neuro.uu.se; Phone: +46-18-4714518; Fax: +46-18-511540.
